# Bupivacaine–fentanyl isobaric spinal anesthesia reduces the risk of ICU admission in elderly patients undergoing lower limb orthopedic surgery

**DOI:** 10.1186/s13018-024-04618-x

**Published:** 2024-03-01

**Authors:** Wenxun Liu, Shuzhen Bao, Jiaxin Chen, Yan Li, Yinghua Gu, Qingshan Ye, Kerong Hai

**Affiliations:** 1 Department of Anesthesiology, People’s Hospital of Ningxia Hui Autonomous Region, 301 Zhengyuan North Street, Yinchuan, 750001 China; 2 Department of Anesthesiology, Ganzhou People’s Hospital, Ganzhou, China; 3 Ningxia Medical University, Yinchuan, China

**Keywords:** Spinal anesthesia, General anesthesia, Lower limb orthopedic surgery, Intensive care unit, Hemodynamics

## Abstract

**Background:**

To evaluate if bupivacaine–fentanyl isobaric spinal anesthesia could reduce the risk of ICU admission compared with general anesthesia in elderly patients undergoing lower limb orthopedic surgery.

**Methods:**

This study comprised a retrospective review of all lower limb orthopedic surgeries performed at our hospital between January 2013 and December 2019. According to anesthesia methods, patients were divided into the spinal anesthesia group (*n* = 1,728) and the general anesthesia group (*n* = 188). The primary outcome evaluated was the occurrence of ICU admission. Secondary outcomes included hemodynamic changes, postoperative complications, and mortality.

**Results:**

Repeated measure analysis of variance indicated that the difference between the two groups in the systolic blood pressure (SBP) was not significant before anesthesia (T0), immediately after anesthesia (T1), and before leaving the operation room (T8) (*P* > 0.05), but significant (*P* < 0.01) from 5 min after anesthesia (T2) to after operation (T7). The proportions of ICU admission (6.4% vs. 23.8%, *P* < 0.01) and unplanned intubation (0.1% vs. 3.8%, *P* < 0.01) were significantly lower in the spinal anesthesia group compared with those in the general anesthesia group. Multivariate logistic regression revealed that after controlling for potential confounding factors, the odds of ICU admission for patients in the spinal anesthesia group was 0.240 times (95% CI 0.115–0.498; *P* < 0.01) than those in the general anesthesia group.

**Conclusions:**

Bupivacaine–fentanyl isobaric spinal anesthesia significantly reduced the risk of ICU admission and unplanned intubation, and provided better intraoperative hemodynamics in elderly patients undergoing lower limb orthopedic surgery.

*Trial registration*: This study has been registered in the Chinese Clinical Trial Registry (ChiCTR2000033411).

## Background

Lower limb fracture represents about 1/3 of all fractures and its incidence is sustained increasing among the elderly in this aging society [1]. It is associated with an increased risk of mortality and disability in normal weight-bearing activities. Lower limb orthopedic surgery poses huge challenges to anesthesiologists as adequate analgesia along with fast motor recovery to ambulate the patient early are required. Spinal anesthesia is the most consistent block of choice as it provides excellent anesthesia and muscle relaxation intraoperatively as well as postoperative analgesia, meanwhile, it could avoid postoperative delays in recovery [2]. Moreover, fewer incidences of common side effects and complications are added advantages [3].

Bupivacaine is a common local anesthetic drug via the intrathecal route for lower limb surgeries to provide effective analgesia and sensory block for the surgery [4]. Opioids like fentanyl have been widely used in the subarachnoid block as adjuvants, aiming to prolong the duration of the block and provide postoperative pain relief while minimizing the use of a high dose of local anesthetic [5]. In addition, it is considered to provide hemodynamic stability [6].

In our clinical practice, we observed that the hemodynamic fluctuations were occasionally prominent in elderly patients after induction of general anesthesia in lower limb orthopedic surgery and a large proportion of patients had to be admitted to the intensive care unit (ICU). Though bupivacaine–fentanyl isobaric spinal anesthesia has been widely applied [7, 8], it is still a lack of evidence to demonstrate its superiority in hemodynamic stability and ICU admission compared with general anesthesia.

The purpose of this retrospective study was to evaluate if bupivacaine–fentanyl isobaric spinal anesthesia could reduce the risk of ICU admission compared with general anesthesia in elderly patients undergoing lower limb orthopedic surgery.

## Methods

### Patients

This study comprised a retrospective review of all lower limb orthopedic surgeries performed at our hospital between January 2013 and December 2019. The inclusion criteria were 1) American Society of Anesthesiology (ASA) grade II-IV; 2) aged ≥ 65 years; 3) Bupivacaine–fentanyl isobaric spinal anesthesia or general anesthesia with endotracheal intubation. Patients were excluded if they had osteofascial compartment syndrome, multiple fractures, severe infections, cancers; or other severe comorbidities.

### Anesthesia

Anesthesia was administered by the usual clinical anesthesia staff. Patients were divided into spinal anesthesia group and general anesthesia group according to anesthesia methods. In the spinal anesthesia group, with the patients in the lateral position, the subarachnoid space was entered at the L2-3, or L3-4 interspace via the midline approach using a 25G Quincke spinal needle. Then, each patient received 3 ml of a solution containing isobaric bupivacaine (15 mg) and fentanyl (50 ug). For patients assigned to general anesthesia, after induction of general anesthesia with propofol, sufentanil, and cisatracurium, providers were instructed to use an intravenous anesthetic agent (propofol or remifentanil) for maintenance, with the choice of agent conforming to their usual practice, and to use an endotracheal tube, supraglottic airway, or another device for airway management in accordance with local practice.

### Data collection and outcomes

Patient’s demographic and clinical variables including age, gender, body mass index (BMI), ASA classification, type of surgery, preoperative comorbidities, length of operation, and length of hospital stay were recorded. Preoperative laboratory tests including White blood cell count (WBC), blood platelet count (BPC), hemoglobin (Hb), serum creatinine (Scr), serum albumin (SA), and international normalized ratio (INR) were also collected.

The primary outcome evaluated was the occurrence of ICU admission. Secondary outcomes included hemodynamic changes, postoperative complications, and mortality. Hemodynamic parameters including systolic blood pressure (SBP), diastolic blood pressure (DBP), and heart rate (HR) were measured before anesthesia (T0), immediately (T1), 5 min (T2), 10 min (T3), 15 min (T4), 30 min (T5), and 60 min (T6) after anesthesia, after the operation (T7), and before leaving the operation room (T8).

### Statistical analysis

Continuous variables with normal distribution are presented as means ± SD and compared with the use of a t test. All categorical variables were summarized and expressed as proportions and compared with the use of the chi-square test with normal approximation or Fisher’s exact test, as appropriate. Repeated measurement data were analyzed by repeated measures one-way analysis of variance (ANOVA), followed by post hoc analyses. Multivariate logistic regression was performed to analyze the association of anesthesia methods with the odds of ICU admission after controlling for potential confounding factors. All tests were 2-sided and a *P* value of less than 0.05 was considered significant.

All statistical analyses were performed with the SPSS statistical software program package (SPSS version 20.0 for Windows, Armonk, NY: IBM Corp.).

## Results

### Patients’ characteristics

Among 2,589 elderly patients undergoing lower limb orthopedic surgeries performed at our hospital, 673 cases were excluded and 1,916 were included in the analysis. These patients were divided into the spinal anesthesia group (*n* = 1,728) or the general anesthesia group according to the anesthesia method (*n* = 188). As shown in Table 1, the proportion of operation time > 1.5 h was significantly lower in the spinal anesthesia group compared with that in the general anesthesia group (*P* < 0.01). The differences in age, gender, BMI, ASA grade, surgery site, comorbidities, smoking, drinking, and laboratory tests were not significant between the two groups (*P* > 0.05).

**Table 1:** Patients’ baseline characteristics and clinical variables

	General anesthesia (*n* = 188)	Spinal anesthesia (*n* = 1,728)	*P* value
Age	74.2 ± 5.8	73.4 ± 6.3	0.06
Gender, *n* (%)			0.17
Male	121 (64.4%)	1,199 (69.4%)	
Female	67 (35.6%)	529 (30.6%)	
BMI	24.10 ± 3.35	24.43 ± 3.92	0.32
ASA grade ≥ III, *n* (%)	119 (63.3%)	1,074 (62.2%)	0.79
Surgery site, *n* (%)			0.37
Hip	84 (44.7%)	699 (40.5%)	
Knee	85 (45.2%)	803 (46.5%)	
Others	19 (10.1%)	226 (13.1%)	
Comorbidities, *n* (%)			
Hypertension	82 (43.6%)	806 (46.6%)	0.43
Diabetes	28 (14.9%)	256 (14.8%)	0.98
Respiratory disease	16 (8.5%)	111 (6.4%)	0.28
Cardiovascular disease	21 (11.2%)	256 (14.8%)	0.64
Stroke	11 (5.9%)	138 (8.0%)	0.30
Smoking, *n* (%)	20 (10.6%)	177 (10.2%)	0.87
Drinking, *n* (%)	5 (2.7%)	54 (3.1%)	0.70
Laboratory tests			
WBC	8.0 ± 3.2	7.6 ± 2.6	0.42
BPC	217.9 ± 72.3	217.5 ± 71.4	0.94
Hb	117.1 ± 19.6	115.2 ± 17.0	0.53
Scr	66.0 ± 26.2	66.0 ± 33.7	0.99
SA	37.1 ± 4.9	37.7 ± 4.3	0.15
INR	1.0 ± 0.2	1.0 ± 0.2	0.12
Operation time > 1.5 h, *n* (%)	160 (85.0%)	1,231 (71.2%)	< 0.01

*BMI* body mass index, *ASA* American Society of Anesthesiology, *WBC* White blood cell count, *PBC* blood platelet count, *Hb* hemoglobin, *Scr* serum creatinine, *SA* serum albumin, *INR* international normalized ratio

### Intraoperative hemodynamic changes

**Fig. 1:**
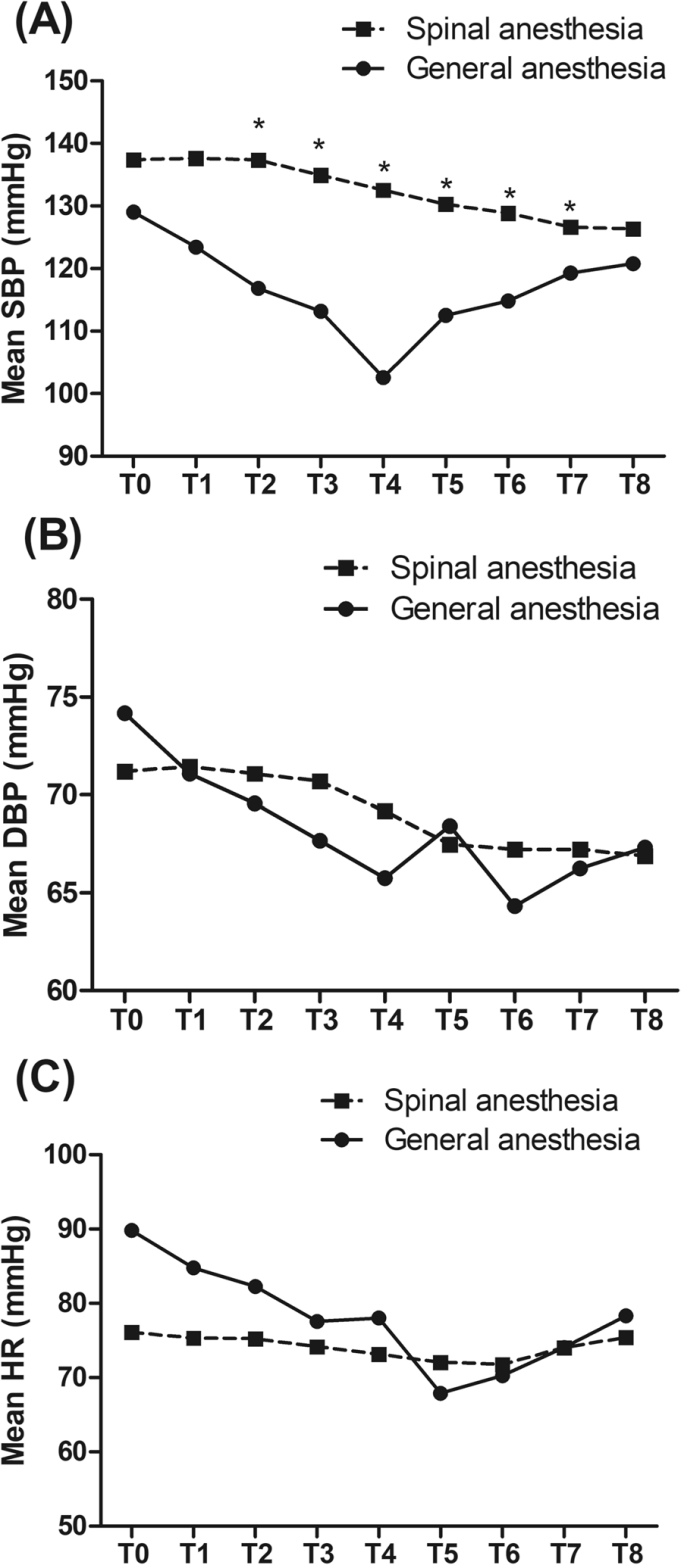
Comparison of hemodynamic parameters including **A** systolic blood pressure (SBP), **B** diastolic blood pressure (DBP), and **C** heart rate (HR) between the spinal anesthesia group and the general anesthesia group. The parameters were measured before anesthesia (T0), immediately (T1), 5 min (T2), 10 min (T3), 15 min (T4), 30 min (T5), and 60 min (T6) after anesthesia, after the operation (T7), and before leaving the operation room (T8). *P < 0.05 compared with the general anesthesia group

Repeated measure ANOVA showed that after controlling for baseline characteristics as covariates, both between- and within-group effects were significant (both *P* < 0.01), and the interaction between time and group was also significant (*P* < 0.01) in the SBP (Fig. 1A), indicating that anesthesia methods influenced the intraoperative hemodynamic changes. *Post hoc* analysis showed that the difference between the two groups in the SBP was not significant at T0, T1, and T8 (*P* > 0.05), but significant (*P* < 0.01) from T2 to T7.

In terms of DBP (Fig. 1B) and HR (Fig. 1C), repeated measure ANOVA indicated that between- and within-group effects as well as the interaction between time and group were not significant.

### ICU admission, mortality, and postoperative complications

As shown in Table 2, the proportions of ICU admission (6.4% vs. 23.8%, *P* < 0.01) and unplanned intubation (0.1% vs. 3.8%, *P* < 0.01) were significantly lower in the spinal anesthesia group compared with those in the general anesthesia group. The incidences of mortality and most complications including pulmonary infection, pulmonary embolism, deep vein thrombosis, blood transfusion, reoperation, and urinary retention did not differ significantly between the two groups (all *P* > 0.05).

**Table 2:** Comparison of ICU admission, mortality, and postoperative complications between general and spinal anesthesia groups

	General anesthesia	Spinal anesthesia	*P* value
ICU admission, *n* (%)	44 (23.8%)	110 (6.4%)	** < 0.01**
Mortality, *n* (%)	1 (0.5%)	3 (0.2%)	0.86
Complications			
Pulmonary infection, *n* (%)	1 (0.5%)	8 (0.5%)	1.00
Pulmonary embolism, *n* (%)	1 (0.5%)	4 (0.2%)	0.99
Deep vein thrombosis, *n* (%)	5 (2.7%)	26 (1.5%)	0.36
Unplanned intubation, *n* (%)	7 (3.8%)	2 (0.1%)	** < 0.01**
Blood transfusion, *n* (%)	13 (7.0%)	90 (5.2%)	0.32
Reoperation, *n* (%)	2 (1.1%)	19 (1.1%)	1.00
Urinary retention, *n* (%)	2 (1.1%)	30 (1.7%)	0.70

Multivariate logistic regression (Table 3) revealed that after controlling for potential confounding factors including age, gender, BMI, ASA grade, surgery site, comorbidities, smoking, drinking, laboratory tests, and operation time, the odds of ICU admission for patients in the spinal anesthesia group was 0.240 times (95% CI 0.115–0.498; *P* < 0.01) than those in the general anesthesia group. In addition, the age ≥ 80 years (OR = 7.219; 95% CI 3.814–13.664; *P* < 0.01), operation time ≥ 1.5 h (OR = 8.346; 95% CI 4.283–16.264; *P* < 0.01) and preoperative diabetes (OR = 3.027; 95% CI 1.420–6.451; *P* < 0.01) were also significantly associated with the risk of ICU admission.

**Table 3:** Multivariate logistic regression analysis for exploring the risk factors of ICU admission

	Odds ratio	95% CI	*P* value
Spinal vs. General anesthesia	0.240	0.115–0.498	< 0.01
Age ≥ 80 years vs. < 80 years	7.219	3.814–13.664	< 0.01
Operation time ≥ 1.5 h vs. < 1.5 h	8.346	4.283–16.264	< 0.01
Preoperative diabetes	3.027	1.420–6.451	< 0.01

## Discussion

Bupivacaine is one of the most widely used drugs for spinal anesthesia. However, the use of bupivacaine alone provides a limited duration of blockade (ranging from 60 to 120 min) and shorter postoperative analgesia. Opioids like fentanyl are commonly used as adjuvants to local anesthetics to prolong the duration of sensory and motor block with better hemodynamic stability [3]. In this retrospective study involving 1,916 older adults undergoing lower limb surgery, the incidences of ICU admission and unplanned intubation were significantly lower in patients assigned to receive bupivacaine–fentanyl isobaric spinal anesthesia compared with those assigned to receive general anesthesia. In addition, the difference between the two groups in the SBP was significant (*P* < 0.01) from T2 to T7. Other secondary outcomes including changes in DBP and HR, most postoperative complications, and mortality did not differ substantially according to anesthesia type.

ICU beds are scarce resources within hospitals, which substantially contribute to the increase in healthcare expenditures. Currently, there is no strict definition regarding the criteria for ICU admission after surgery and the decision mainly depends on the physician’s perception. Indeed, several intraoperative factors might affect the decision. Firstly, in line with many reports that spinal anesthesia has been reported to reduce the requirement of postoperative mechanical ventilation [9], our study shows that spinal anesthesia significantly reduced the incidence of unplanned intubation, indicating that it has little effect on pulmonary function. In addition, consistent with many randomized controlled trials [10, 11], our results indicated that spinal anesthesia resulted in improved intraoperative hemodynamic stability. Moreover, some study [12] argued that spinal anesthesia may provide intraoperative benefits that may affect the patient’s overall physical status at the conclusion of surgery, and influence a physician’s decision to admit the patient to the ICU.

Limitations of this study include its retrospective design and findings. Some preoperative and postoperative parameters might be missing. In addition, the single-center design may limit its dissemination. Moreover, the sample size in the spinal anesthesia group is much larger than that in the general anesthesia group. This heterogeneity may have limited our ability to detect differences in outcomes between the groups.

## Conclusions

In conclusion, in this retrospective study involving older patients undergoing lower limb surgery, spinal anesthesia significantly reduced the risk of ICU admission and unplanned intubation and provided better intraoperative hemodynamics.

## Data Availability

The datasets used and/or analyzed during the current study are available from the corresponding author upon reasonable request.
